# Prepregnancy and early pregnancy calcium supplementation among women at high risk of pre-eclampsia: a multicentre, double-blind, randomised, placebo-controlled trial

**DOI:** 10.1016/S0140-6736(18)31818-X

**Published:** 2019-01-26

**Authors:** G Justus Hofmeyr, Ana Pilar Betrán, Mandisa Singata-Madliki, Gabriela Cormick, Stephen P Munjanja, Susan Fawcus, Simpiwe Mose, David Hall, Alvaro Ciganda, Armando H Seuc, Theresa A Lawrie, Eduardo Bergel, James M Roberts, Peter von Dadelszen, José M Belizán, Calcium and Pre-eclampsia Study Group, Fernando Althabe, José M Belizán, Eduardo Bergel, Alvaro Ciganda, Gabriela Cormick, Diane Sawchuck, Marianne Vidler, Saadiqa Allie, John Anthony, Karlyn Frank, Annmarie de Greeff, Sue Fawcus, David Hall, Justus Hofmeyr, Mvuseleli Kovane, Patience Kovane, Theresa Lawrie, Simpiwe Mose, Nolundi Mshweshwe, Velisa Mqikela, Pamela Njikelana, Natalia Novikova, Adegboyega Oyebajo, Catherine Parker, Angel Phuti, Mandisa Singata-Madliki, Erika van Papendorp, Xoliswa Williams, Ana Pilar Betrán, Tina Dannemann, Armando Seuc, Laura Magee, Peter von Dadelszen, France Donnay, Sharla Drebit, Jim Roberts, Bothwell Guzha, Emilia Makaza, Sarah Manyame, Stephen Munjanja, Eunice Tahuringana

**Affiliations:** a Effective Care Research Unit, Eastern Cape Department of Health, Universities of the Witwatersrand, Walter Sisulu, and Fort Hare, East London, South Africa; b HRP–UNDP–UNFPA–UNICEF–WHO–World Bank Special Programme of Research, Development and Research Training in Human Reproduction, Department of Reproductive Health and Research, WHO, Geneva, Switzerland; c Department of Mother and Child Health Research, Institute for Clinical Effectiveness and Health Policy (IECS-CONICET), Buenos Aires, Argentina; d Department of Human Biology, Faculty of Health Sciences, University of Cape Town, South Africa; e Department of Obstetrics and Gynaecology, University of Cape Town, South Africa; f University of Zimbabwe, Harare, Zimbabwe; g Department of Obstetrics and Gynaecology, Chris Hani Baragwanath Hospital, University of the Witwatersrand, Johannesburg, South Africa; h Department of Obstetrics and Gynaecology, Stellenbosch University and Tygerberg Hospital, Cape Town, South Africa; i Magee-Women's Research Institute, Department of Obstetrics and Gynecology, Epidemiology and Clinical and Translational Research, University of Pittsburgh, Pittsburgh, PA, USA; j Department of Women and Children's Health, School of Life Course Sciences, King's College London, London, UK

## Abstract

**Background:**

Reducing deaths from hypertensive disorders of pregnancy is a global priority. Low dietary calcium might account for the high prevalence of pre-eclampsia and eclampsia in low-income countries. Calcium supplementation in the second half of pregnancy is known to reduce the serious consequences of pre-eclampsia; however, the effect of calcium supplementation during placentation is not known. We aimed to test the hypothesis that calcium supplementation before and in early pregnancy (up to 20 weeks' gestation) prevents the development of pre-eclampsia

**Methods:**

We did a multicountry, parallel arm, double-blind, randomised, placebo-controlled trial in South Africa, Zimbabwe, and Argentina. Participants with previous pre-eclampsia and eclampsia received 500 mg calcium or placebo daily from enrolment prepregnancy until 20 weeks' gestation. Participants were parous women whose most recent pregnancy had been complicated by pre-eclampsia or eclampsia and who were intending to become pregnant. All participants received unblinded calcium 1·5 g daily after 20 weeks' gestation. The allocation sequence (1:1 ratio) used computer-generated random numbers in balanced blocks of variable size. The primary outcome was pre-eclampsia, defined as gestational hypertension and proteinuria. The trial is registered with the Pan-African Clinical Trials Registry, number PACTR201105000267371. The trial closed on Oct 31, 2017.

**Findings:**

Between July 12, 2011, and Sept 8, 2016, we randomly allocated 1355 women to receive calcium or placebo; 331 of 678 participants in the calcium group versus 320 of 677 in the placebo group became pregnant, and 298 of 678 versus 283 of 677 had pregnancies beyond 20 weeks' gestation. Pre-eclampsia occurred in 69 (23%) of 296 participants in the calcium group versus 82 (29%) of 283 participants in the placebo group with pregnancies beyond 20 weeks' gestation (risk ratio [RR] 0·80, 95% CI 0·61–1·06; p=0·121). For participants with compliance of more than 80% from the last visit before pregnancy to 20 weeks' gestation, the pre-eclampsia risk was 30 (21%) of 144 versus 47 (32%) of 149 (RR 0·66, CI 0·44–0·98; p=0·037). There were no serious adverse effects of calcium reported.

**Interpretation:**

Calcium supplementation that commenced before pregnancy until 20 weeks' gestation, compared with placebo, did not show a significant reduction in recurrent pre-eclampsia. As the trial was powered to detect a large effect size, we cannot rule out a small to moderate effect of this intervention.

**Funding:**

The University of British Columbia, a grantee of the Bill & Melinda Gates Foundation; UNDP–UNFPA–UNICEF–WHO–World Bank Special Programme of Research, Development and Research Training in Human Reproduction, WHO; the Argentina Fund for Horizontal Cooperation of the Argentinean Ministry of Foreign Affairs; and the Centre for Intervention Science in Maternal and Child Health.

## Introduction

Hypertension is estimated to complicate 5% of all pregnancies and 11% of first pregnancies, and half of these cases are associated with pre-eclampsia (gestational hypertension plus proteinuria).^1^ Hypertensive disorders of pregnancy are the direct cause of death of about 30 000 women annually,^2^ or approximately 14% of maternal deaths,^3^ most of which occur in low-income countries. In a survey of 29 countries in Africa, Asia, Latin America, and the Middle East, 25·9% of women with severe maternal outcomes (ie, maternal death or near miss death) had pre-eclampsia or eclampsia, which was the direct cause of 20% of reported maternal deaths in these country settings.^4^ Therefore, reducing maternal mortality and morbidity from hypertensive disorders is a global priority.

**Research in context**

**Evidence before this study**

We searched PubMed for articles published in English between Jan 1, 1980, and Dec 31, 2017, reporting the effect of prepregnancy or early pregnancy calcium supplementation on pre-eclampsia. Among 185 records, we identified one randomised controlled trial with 60 participants that evaluated a calcium-containing antioxidant supplement commenced at 8 to 12 weeks' gestation compared with placebo. Participants were pregnant women who screened positive for low antioxidant status. This small study reported reductions in pre-eclampsia (two of 29 *vs* nine of 31) and miscarriage (none of 29 *vs* eight of 31) in favour of the intervention. However, as the intervention comprised multiple nutrients and the study was underpowered to detect a difference in pre-eclampsia, the findings with respect to calcium supplementation in early pregnancy are difficult to interpret. Evidence from a Cochrane systematic review of randomised trials shows that calcium supplementation after 20 weeks of pregnancy reduces the serious consequences of pre-eclampsia and the prevalence of pre-eclampsia. The effect was mostly shown in the smaller trials contributing to the review, whereas in a large WHO-led trial it was not significant. We hypothesised that, as the pathological process of pre-eclampsia might evolve early on in pregnancy, calcium supplementation from 20 weeks' gestation could be too late to have a substantial effect on this condition.

**Added value of this study**

This study is the first randomised trial to our knowledge to report the effects of calcium supplementation before and in early pregnancy, when pre-eclampsia is thought to develop. Although our primary findings on pre-eclampsia are not significant, they might justify further research into the relation between early pregnancy calcium intake and pre-eclampsia.

**Implications of all the available evidence**

The overall benefits of calcium supplementation throughout pregnancy would be the sum of any prepregnancy and early pregnancy effects plus those shown in previous studies of calcium supplementation after 20 weeks' gestation. Findings from our study, with appropriate recognition of their absence of significance, should be considered together with all other available evidence on the benefits of adequate calcium intake for pregnant women and the general population, in determining the cost and benefits of strategies to improve calcium intake at a population level.

Although pre-eclampsia is considerably more prevalent in disadvantaged than advantaged populations, two striking exceptions have been identified. Over 50 years ago, a low prevalence of pre-eclampsia was reported in Ethiopia, where the local diet contained high levels of calcium.^5^ Similarly, in 1980 it was observed that among Mayan Indians in Guatemala, who traditionally soaked their staple corn in lime (calcium hydroxide) water before cooking, pre-eclampsia was uncommon.^6^ These observations stimulated research interest in the concept that the link between pre-eclampsia and poverty might be a dietary deficiency of calcium.

The hypothesis that calcium supplementation during pregnancy might reduce the prevalence of pre-eclampsia was tested in several randomised controlled trials (RCTs) beginning in the late 1980s.^7^, ^8^, ^9^, ^10^, ^11^, ^12^, ^13^, ^14^, ^15^ In 2001, WHO did a large RCT (n=8325) of calcium supplementation (1·5 g per day) from 20 weeks' gestation among pregnant women with low calcium intake.^16^ Although the supplement did not prevent pre-eclampsia (risk ratio [RR], 0·91; 95% CI, 0·69–1·19), it reduced its severity (0·76; 0·66–0·89), severe maternal morbidity and mortality (0·80; 0·70–0·91), and neonatal mortality (0·70; 0·56–0·88).^16^

A meta-analysis of 13 RCTs in a Cochrane systematic review found that calcium supplementation of at least 1 g daily from mid-pregnancy (20 weeks) was associated with a 55% reduction in pre-eclampsia.^17^ However, the magnitude of this effect was thought to be over-estimated owing to the small study effects.^17^ The review highlighted that the reduction in pre-eclampsia was greatest in the subgroup of women at high risk of pre-eclampsia, and those with low dietary calcium intake. It also found a reduction in the composite outcome of maternal death or serious morbidity with calcium supplementation.

A nested study within the large WHO trial of calcium supplementation during the second half of pregnancy (ie, >20 weeks) failed to show an effect on biochemical measures commonly elevated in pre-eclampsia (ie, serum urate, platelet count, and urine protein:creatinine ratio).^18^ This absence of effect was consistent with the findings of no difference in proteinuria in the main WHO trial (8312 participants, RR 1·01; 95% CI 0·88–1·15).^16^ We hypothesised that calcium supplementation in the second half of pregnancy reduces blood pressure, and thus the diagnosis and severe manifestations of pre-eclampsia, without having a substantial effect on the underlying pathology (eg, defective placentation in early pregnancy). If our hypothesis is correct, an inadequate calcium intake before and during early pregnancy might place certain populations at higher risk of pre-eclampsia.

To our knowledge, no randomised trials have specifically evaluated the effect of calcium supplementation commenced before pregnancy on pre-eclampsia. In this paper, we report the findings of a randomised placebo-controlled trial among women who had pre-eclampsia or eclampsia in their previous pregnancy, designed to establish whether calcium supplementation started before pregnancy and continued during the first half of pregnancy might reduce the subsequent development of pre-eclampsia and other adverse outcomes, despite high dose supplementation to both groups after 20 weeks' gestation.

## Methods

### Study design

This was a multicentre, parallel arm, double blind, randomised, placebo-controlled trial, done in South Africa, Zimbabwe, and Argentina. In South Africa (Frere and Cecilia Makiwane Hospitals in East London; Chris Hani Baragwanath Hospital Johannesburg; Groote Schuur, Mowbray Maternity and Tygerberg Hospitals in Cape Town) and Zimbabwe (Harare Maternity and Mbuya Nehanda Maternity Hospitals, Harare) sites were state secondary or tertiary referral hospitals with large obstetric services (4000 to 20 000 births per year) providing comprehensive obstetric care, serving urban and rural lower-income populations. The sites in Argentina comprised three maternity hospitals, one in the province of Tucumán and two in Buenos Aires: the Institute of Maternity and Gynecology of Our Lady of Mercedes is located in the capital city of Tucumán province and it is the public referral maternity hospital of northwest Argentina with around 9000 deliveries each year; Hospital Italiano and CEMIC are private third-level maternity hospitals in Buenos Aires, each with around 2000 deliveries per year.

The protocol of this study was approved by the Research Project Review Panel of the UNDP–UNFPA–UNICEF–WHO–World Bank Special Programme of Research, Development and Research Training in Human Reproduction at the Department of Reproductive Health and Research of WHO, and the WHO Research Ethics Review Committee, Geneva, Switzerland. Ethical approval was obtained from the appropriate national and institutional ethics review bodies applicable to each study site before the start of the trial. Data management procedures were compliant with good clinical practice.

The protocol of this trial is published online in *The Lancet* (protocol 11PRT/4028).^19^

### Participants

We used several approaches to identify potentially eligible women: direct searching of hospital records; prospective identification of women with, or after, pregnancies complicated by pre-eclampsia or eclampsia to recruit them at a future date; and raising community awareness through posters, community outreach visits, and radio interviews. Details of recruitment and retention strategies are reported elsewhere.^20^

Participants were parous women whose most recent pregnancy had been complicated by pre-eclampsia or eclampsia and who were intending to become pregnant. All participants provided informed written consent. Women were not eligible for the trial if they were younger than 18 years old; were already pregnant; were taking calcium supplementation; had chronic hypertension with persistent proteinuria; had a history or symptoms of urolithiasis, renal disease, or parathyroid disease; were not in a sexual relationship; were using long-term contraception (eg, hormonal injections or implant, intrauterine contraceptive device, or sterilisation); or were unwilling to give informed consent.

### Randomisation and masking

The random allocation sequence was generated centrally at WHO headquarters in Geneva, Switzerland, using computer-generated random numbers in a ratio of 1:1 and in balanced blocks of variable size, stratified by site. Participants, care providers, and outcome assessors were all masked to group allocation. Calcium and placebo tablets were packed in identical 12-week treatment bottles, each containing 84 tablets. Participants were randomly assigned to a calcium supplementation or placebo group. The calcium group received one chewable tablet containing 500 mg elemental calcium (as calcium carbonate) daily from prepregnancy randomisation until 20 weeks' gestation. The placebo tablet was indistinguishable from the calcium tablet in appearance and taste. At enrolment, allocation was done using an online service hosted by WHO, which allocated the next available treatment pack number from the site's supply. At subsequent visits, the same online service was used to allocate an appropriate pack number, which ensured continuation of the allocated treatment without revealing group allocation.

After 20 weeks' gestation, all participants received open-label calcium tablets. Three bottles of 84 tablets each were supplied at a time, which was sufficient for a 12-week period (ie, from 20 weeks' to 32 weeks' gestation and from 32 weeks' gestation until birth).

### Procedures

The trial methods built on those used in the WHO trial of calcium supplementation during the second half of pregnancy that was done among pregnant women in similar settings,^16^ incorporating experiences and lessons learnt.

Participants were asked to chew their allocated tablet in the evening at a time distant from taking food or iron supplements. They were encouraged not to take any additional calcium supplements. For those participants who needed analgesics, paracetamol was recommended, and for those needing antacids, a non-calcium-based antacid was recommended.

The rationale for the calcium dosage selected was based on a dietary calcium survey done to inform the design of the WHO trial of calcium supplementation during the second half of pregnancy,^16^ which showed that pregnant primiparous women in Argentina had a median daily dietary calcium intake of 481 mg and in South Africa a median daily dietary calcium intake of 567 mg.^20^ Supplementation with 500 mg of calcium per day could thus achieve a median daily intake of about 1000 mg. This amount would approximate the daily calcium intake among pregnant women in high-income countries.^21^ Moreover, 500 mg is a level of supplementation that is achievable with food fortification.^22^

In addition to receiving the allocated calcium or placebo tablets until 20 weeks' gestation, all participants also received calcium supplementation as recommended by WHO (1·5 g elemental calcium daily) from 20 weeks' gestation until childbirth.^23^

Once recruited, participants were asked to return to the clinic-based research office every 12 weeks until pregnant, and then every 12 weeks until childbirth for follow-up visits. The visits during pregnancy were scheduled to take place as close as possible to 8 weeks', 20 weeks', and 32 weeks' gestation, alongside the routine antenatal care visits. Contact was maintained by 4-weekly telephone calls.

After participants had given birth, members of the research team extracted their outcome data from hospital records. At 6 weeks after childbirth, participants were telephoned to enquire about any complications since discharge from hospital. Participants who entered the trial and did not become pregnant were followed up for 1 month after cessation of calcium supplements.

Data were recorded on case report forms specifically designed for the trial and entered in duplicate in a web-based data management system (OpenClinica). Blood pressure was recorded using mercury sphygmomanometers at the African sites, and with electronic blood pressure monitors in Argentina. All members of the research team were trained and assessed on their ability to measure blood pressure, with the British Hypertension Society training materials. Blood pressure measurements were standardised in the following way: participants were at rest, having been seated for 5 min or in bed with their abdomen tilted at least 30° to the left; the cuff was placed on the right arm at the level of the heart; two blood pressure measurements (systolic and diastolic) were taken at 3-min intervals; diastolic blood pressure was measured at the fifth Korotkoff sound (disappearance of the sounds); and the average of the two readings was recorded.

This was a pragmatic trial and, therefore, pre-eclampsia was recorded as an outcome if it was diagnosed by a health-care provider and recorded in the participant's medical records or if we identified both high blood pressure and proteinuria, as per the trial definitions of pre-eclampsia, in a participant's medical records.

Urine was collected routinely from all participants during trial antenatal visits, on admission in labour, or before elective caesarean section, to detect the presence of protein with a dipstick. This process was in accordance with routine clinical practice at the study sites.

Results of relevant laboratory investigations during the provision of routine care, such as blood haemoglobin concentrations, were recorded in the case report forms, and for those who developed pre-eclampsia, these usually included a blood platelet count, serum urate, urea, creatinine, and liver function tests (ie, lactate dehydrogenase, alanine aminotransferase and aspartate aminotransferase, and a urine protein:creatinine ratio, urine protein, or 24 h urinary protein investigation, or both). Where several tests had been done, the last test before labour or caesarean section was used or, if none had been done previously, the first test after the start of labour or caesarean section was used.

In a substudy done at all the African sites, data on dietary intake were obtained from participants at 20 weeks' gestation, using a triple pass 24 h dietary recall questionnaire.^24^ A detailed description of the methodology and analysis of these findings has been published elsewhere.^25^ Diet assessment at 20 weeks' gestation showed a mean daily calcium intake of 441 mg per day (SD 87·7; n=224) in South African women and 360·5 mg per day (SD 171·4; n=88) in Zimbabwean women, confirming the very low dietary calcium intake among trial participants.

Every 12 weeks during trial follow-up visits both before and during pregnancy, all returned, unused tablets were counted, and the number assumed to have been taken was calculated and recorded. Treatment compliance was calculated by dividing the number of used tablets by the total number of tablets that should have been taken since the last count. A new bottle of 84 tablets was supplied to participants and returned tablets were stored until the end of the trial.

### Outcomes

The original protocol registered with the Pan-African Clinical Trials Registry identified three primary outcomes: pre-eclampsia, pre-eclampsia or pregnancy loss before labour at any gestation, and severe maternal morbidity and mortality index: defined as one or more of the specified secondary outcomes. The reason for the second primary outcome was to account for possible confounding of the pre-eclampsia outcome by pregnancy loss, which might be causally related. Before trial commencement, we revised the protocol to specify only the first primary outcome.

Our primary outcome was therefore pre-eclampsia, defined as gestational hypertension and proteinuria (see the panel for definitions). A full list of secondary outcomes can be found in the panel.

Panel **Outcomes and definitions**

**Primary outcome:**

• Pre-eclampsia, defined as gestational hypertension and gestational proteinuria, as defined below, or diagnosed by the attending clinicians

**Secondary outcomes:**

• Pre-eclampsia or pregnancy loss, or both, at any gestational age
• Gestational hypertension (diastolic blood pressure >90 mm Hg on two occasions 4 h apart; or >110 mm Hg once, or systolic blood pressure >140 mm Hg on two occasions 4 h apart; or both, or >160 mm Hg once, after 20 weeks' gestation)
• Gestational proteinuria (2 or more on urine dipstick, or >300 mg/24 h, or >500 mg/L or urinary protein: creatinine ratio >0·034 g/mmol, after 20 weeks' gestation
• Pregnancy loss at any gestational age, including miscarriage and stillbirth, excluding requested abortion
• No pregnancy during study period
• Severe gestational hypertension (systolic blood pressure >160 mm Hg on two occasions 4 h apart, or once followed by antihypertensive therapy, or diastolic blood pressure >110 mm Hg on two occasions 4 h apart, or once followed by antihypertensive therapy, after 20 weeks' gestation)
• Early onset pre-eclampsia (<32 weeks' gestation)
• Severe pre-eclampsia (proteinuria plus severe diastolic [>110 mm Hg] or systolic [>160 mm Hg] hypertension)
• Moderately severe thrombocytopenia (<100 × 10^9^/L)
• Uric acid more than reference values for gestational age
• Renal failure (creatinine >120 mmol/L)
• Liver failure (aspartate aminotransferase >70 U/L)
• Eclampsia
• Placental abruption
• Pulmonary oedema
• Stroke
• Intensive care unit admission >24 h
• HELLP syndrome
• Maternal death
• Participant hospital stay ≥7 days after childbirth
• Caesarean section
• Birthweight <2500 g
• Preterm birth (<37 weeks' gestation)
• Early preterm birth (<32 weeks' gestation)
• Apgar score <7 at 5 min
• Perinatal death or admission to neonatal intensive care unit for 24 h or more
• Stillbirth
• Pregnancy loss, stillbirth, or neonatal death before discharge
• Pregnancy loss, stillbirth, or neonatal death before 6 weeks
• Previous WHO calcium trial composites (severe pre-eclamptic complications index* and severe maternal morbidity and mortality index†) and compliance outcomes (proportion of expected tablet intake based on counts of returned tablets)^4^

HELLP syndrome=haemolysis, elevated liver enzymes, and low platelet count. *Severe pre-eclamptic complications index: severe pre-eclampsia, early-onset pre-eclampsia (<32 weeks' gestation), eclampsia, HELLP syndrome, placental abruption, or severe gestational hypertension. †Severe maternal morbidity and mortality index: maternal admission to intensive care, eclampsia, severe pre-eclampsia, placental abruption, HELLP syndrome, renal failure, or death.

### Statistical analysis

The sample size calculation was informed by the WHO trial of calcium supplementation in the second half of pregnancy, where hypertension occurred in 14% of the relatively low-risk participants who received calcium supplementation from 20 weeks' gestation.^16^ The risk of recurrent pre-eclampsia for women with previous pre-eclampsia or eclampsia is estimated to be at least 25%.^26^ We calculated that, to show a reduction in pre-eclampsia from 25% to 15%, we would need 540 participants with pregnancies continuing beyond 20 weeks' gestation (0·05 α level, 80% power; calculated using Epi Info software (CDC, Atlanta, GA, USA). We anticipated that 50% of participants recruited would become pregnant during the trial. Allowing for a miscarriage rate of 15% and loss to follow-up of 10%, we needed to recruit approximately 1440 participants who were not pregnant. Enrolment would be stopped once we were able to predict reaching our primary sample size (ie, the 540 participants with pregnancies continuing beyond 20 weeks' gestation).

Analyses of the effects of the intervention were intention to treat (ITT), irrespective of supplementation compliance. We prepared a table of baseline characteristics of the study groups for all participants enrolled and also for those in the final study sample (participants ≥20 weeks' gestation) to make sure that exclusion of those who did not have a pregnancy until 20 weeks did not compromise randomisation. Among those lost to follow-up, the number of participants lost, and their baseline characteristics, were compared to exclude any imbalances between study groups (data not shown). We also did a prespecified per-protocol analysis for participants for whom compliance with calcium supplementation was more than 80%. Other prespecified subgroup analyses are still to be done and will be reported in a separate paper.

For categorical variables, we calculated the number of participants, number of missing values and percentages, and for continuous variables, we calculated the number of participants, number of missing values, minima, maxima, means, and SDs. Categorical variables were compared as risk ratios (RR) with 95% CIs.

In this study, randomisation took place before conception, yet exposure to the primary outcome was limited to those who conceived and carried a pregnancy continuing beyond 20 weeks' gestation during the study period. Therefore, we anticipated potential confounders by first comparing the rates of pregnancy between study groups, for which we used a χ^2^ test. Had a difference in pregnancy rates been found, all subsequent analyses would have been done with the total enrolment numbers for each group as denominators, as well as with the numbers who became pregnant as denominators, and the effect of the differential rates of pregnancy on the results explored. A more likely confounder anticipated was early pregnancy loss, as the same pathology might cause both early pregnancy loss and pre-eclampsia. Any reduction in early pregnancy loss owing to calcium supplementation might increase the proportion of participants diagnosed with pre-eclampsia. For this reason we included the composite secondary outcome, pre-eclampsia or pregnancy loss, or both.

SPSS (version 22) and R (version 3.3.0) were used for all statistical analyses. All tests were done using the usual two-sided, 5% significance levels. The study was overseen by the Data Safety and Monitoring Committee.

The trial was registered with the Pan-African Clinical Trials Registry on Dec 6, 2010, registration number PACTR201105000267371.

### Role of the funding source

The funders of the trial had no role in the trial design, data collection, data analysis, data interpretation, or writing of the report. The primary author, GJH, has full access to all the data in the trial and had the final responsibility for the decision to submit for publication. Midway through the study, the study team was approached by Alternative Discovery & Development, GlaxoSmithKline Medicines Research Centre, UK, who partnered with us to collect blood samples from a sub-group of participants in our trial for an independent, open-innovation pre-eclampsia biomarker study, following a separate protocol which was approved by the trial ethics committee. Apart from direct funding to the largest site (Chris Hani Baragwanath Hospital) specifically for the costs of this blood sample collection, GSK provided no funding to the main trial and did not participate in any aspect of the main trial.

## Results

Between July 12, 2011, and Sept 8, 2016, 2563 women were screened, 1355 were recruited and randomly assigned (678 calcium, 677 placebo), and 651 became pregnant (331 calcium, 320 placebo), among whom 581 had a pregnancy continuing beyond 20 weeks' gestation (298 calcium, 283 placebo). Two participants in the calcium group were lost to follow up after 20 weeks' gestation; therefore, 579 participants contributed data to the final outcome analyses (296 calcium, 283 placebo). Eight participants who did not meet all eligibility criteria were randomly assigned in error; these patients were included in the analysis. Four of them had a pregnancy beyond 20 weeks' gestation two in each group (figure). The trial closed on Oct 31, 2017. Among the 581 participants with pregnancies continuing beyond 20 weeks' gestation, 26 were in Argentina, 160 in Zimbabwe, and 395 in South Africa.

**Figure:**
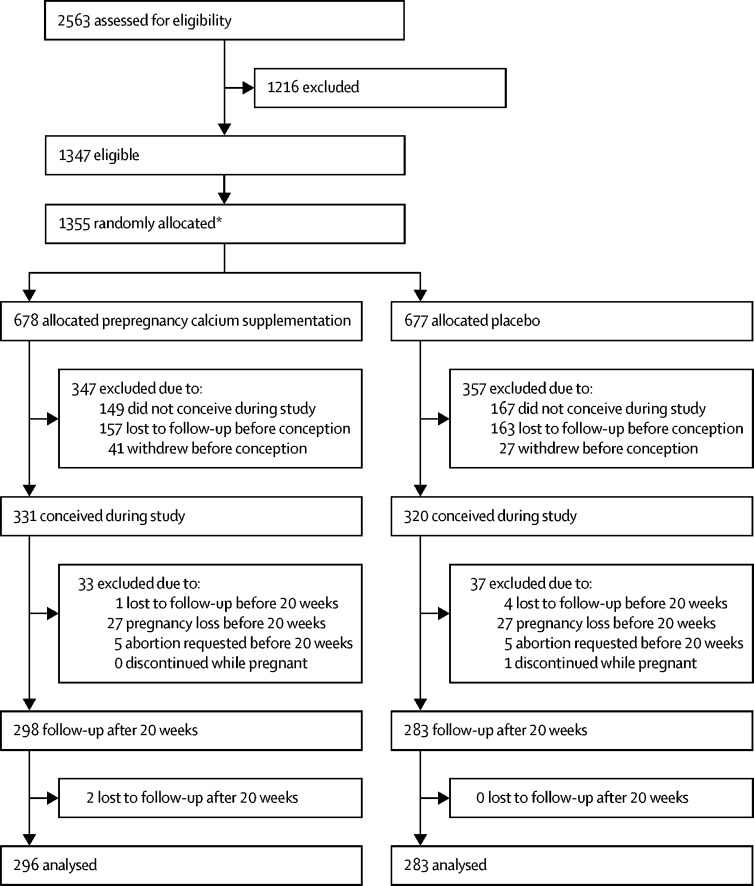
Study profile *Eight subjects were randomised but not eligible.

Baseline characteristics of participants were similar in the two study groups at trial entry (table 1). The largest discrepancy was in previous severe pre-eclampsia, which was assumed to be a chance discrepancy among many parameters, as the randomisation procedure was robust. Baseline characteristics were similar for the final sample, suggesting that randomisation was not compromised by discrepancies between groups comprising the final sample (table 1).

**Table 1:** Baseline characteristics of participants at trial entry (pre pregnancy sample) and of the final sample (pregnancy ≥20 weeks' gestation) according to study group

	**All participants at trial entry (n=1355)**		**Participants with pregnancy ≥20 weeks' gestation (n=581)**	
	Calcium (n=678)	Placebo (n=677)	Calcium (n=298)	Placebo (n=283)
Maternal age, years	678 (30·2) [5·8]	677 (30·4) [5·9]	298 (29·4) [5·4]	283 (29·2) [5·1]
Maternal age <20 years	10/678 (2%)	14/677 (2%)	6/298 (2%)	5/283 (2%)
Maternal weight, kg	649/678 (76·2) [18·5]	641/677 (76·9) [18·8]	289/298 (75·7) [17·6]	276/283 (75·7) [17·4]
Maternal height, cm	617/678 (160·2) [6·3]	613/677 (160) [6·7]	277/298 (160·4) [6·2]	260/283 (159·9) [6·5]
Body mass index, kg/m^2^	610/678 (29·7) [7·1]	608/677 (30·1) [6·9]	273/298 (29·5) [6·8]	258/283 (29·6) [6·6]
Body mass index >30 kg/m^2^	263/610 (43%) [43·1]	268/608 (44%) [44·1]	114/273 (42%) [41·8]	109/259 (42%) [42·1]
Systolic blood pressure at randomisation, mm Hg	675/678 (127·0) [19·3]	674/677 (127·3) [19·2]	297/298 (126.0) [17·3]	283/283 (126·1) [18·8]
Diastolic blood pressure at randomisation, mm Hg	675/678 (81·6) [14·1]	674/677 (82·3) [13·9]	297/298 (81·2) [12·8]	283/283 (81·7) [13·7]
Any health complaint at randomisation	20/678 (3%)	23/677 (3%)	5/298 (2%)	6/283 (2%)
Previous severe pre-eclampsia*	309/403 (77%)	343/414 (83%)	151/188 (80%)	152/178 (85%)
Previous eclampsia	107/643 (17%)	103/639 (16%)	51/284 (18%)	53/266 (20%)
Previous HELLP syndrome	69/561 (12%)	78/561 (14%)	34/251 (14%)	40/229 (18%)
Previous livebirth	349/678 (52%)	337/677 (50%)	129/298 (43%)	118/283 (42%)

Data are in n/N (%) or n/N (mean [SD]). HELLP=haemolysis, elevated liver enzymes, and low platelet count.

* Defined as systolic blood pressure ≥160 mm Hg or diastolic blood pressure ≥110 mm Hg.

Compliance data were available for 421 (73%) of 581 participants from recruitment until the last visit before pregnancy, and for 543 (93%) of 581 of women from the last visit before pregnancy until 20 weeks' gestation (table 2). For those with data available, about half the participants took at least 80% of the expected tablets (53% calcium, 55% placebo) from the last visit before pregnancy to 20 weeks' gestation. In our analysis plan, we regarded this period as most relevant to the trial intervention.

**Table 2:** Compliance of participants in the final sample (pregnancy ≥20 weeks' gestation)

	**Calcium (n=298) [n/N (%)]**	**Placebo (n=283) [n/N (%)]**	**Risk ratio (95% CI) [n/N (%)]**	**p value**
**Compliance >80%**				
From randomisation up to the last visit before pregnancy*	100/213 (47%)	100/208 (48%)	0·98 (0·80–1·19)	0·817
From last visit before pregnancy to the 20-week visit†	145/274 (53%)	149/269 (55%)	0·96 (0·82–1·12)	0·563
**Compliance >50%**				
From randomisation up to the last visit before pregnancy*	173/213 (81%)	187/208 (90%)	0·90 (0·83–0·98)	0·011
From last visit before pregnancy to the 20-week visit†	212/274 (77%)	218/269 (81%)	0·95 (0·88–1·04)	0·292

Data are n/N (%), unless otherwise specified.

* For women with no visit before pregnancy because they became pregnant before 3 months after admission, we have used the admission visit as surrogate.

† For women with no pregnancy visit at 20 weeks' gestation (n=63) the data from the nearest visit to 20 weeks was used: as first option the 32-week visit (ten cases) and as a second option the 8-week visit (15 cases).

None of the pregnancy outcomes were significantly different between groups (table 3).

**Table 3:** Pregnancy outcomes for all participants enrolled (n=1355) according to study group

	**Calcium group (n=678)**	**Placebo group (n=677)**	**p value**
No pregnancy	347 (51%)	357 (53%)	0·567
Pregnancy loss <20 weeks' gestation*	32 (5%)	32 (5%)	1·000
Pregnancy ≥20 weeks' gestation	298 (44%)	283 (42%)	0·420
Livebirth	249 (37%)	228 (34%)	0·240

* Data include five participants in each group who elected to have an abortion.

For the primary outcome, the prevalence of pre-eclampsia was lower in the calcium group (69 [23%] of 296 *vs* 82 [29%] of 283, a reduction of 20%); however, the difference was not significant (RR 0·80 [95% CI 0·61–1·06]; p=0·121; table 4). There were no significant differences in secondary outcomes. The prevalence of pre-eclampsia or pregnancy loss, or both, was 33% in the calcium group and 41% in the placebo group (RR 0·82 [95% CI 0·66–1·00]; p=0·050), which was of borderline significance. There were no significant differences in the composite outcomes defined in the previous WHO trial on calcium supplementation,^16^ namely severe maternal morbidity and mortality index and severe pre-eclamptic index. Per-protocol analysis done for participants with compliance of more than 80% from the last visit before pregnancy to 20 weeks' gestation was consistent with the main trial findings on pre-eclampsia, with fewer participants in the calcium group (30 (21%) of 144) than in the placebo group (47 [32%] of 149) developing pre-eclampsia (RR 0·66, CI 0·44–0·98; p=0·037, table 5). The other pre-specified subgroup and exploratory analyses will be reported separately.

**Table 4:** Primary and secondary outcomes according to study group

	**Calcium group (n/N [%])**	**Placebo group (n/N [%])**	**RR (95% CI)**	**p value**
Pre-eclampsia (primary outcome)	69/296 (23%)	82/283 (29%)	0·80 (0·61–1·06)	0·121
Pre-eclampsia (as a proportion of all women randomised)	69/678 (10%)	82/677 (12%)	0·84 (0·62–1·14)	0·258
Pre-eclampsia and or pregnancy loss at any gestation*	107/323 (33%)	126/310 (41%)	0·82 (0·66–1·00)	0·050
Gestational hypertension	194/296 (66%)	197/283 (70%)	0·94 (0·84–1·05)	0·296
Gestational proteinuria	78/296 (26%)	90/283 (32%)	0·83 (0·64–1·07)	0·149
Pregnancy loss at any gestation*	58/323 (18%)	67/310 (22%)	0·83 (0·61–1·14)	0·248
No pregnancy during the study period†	347/678 (51%)	357/677 (53%)	0·97 (0·88–1·08)	0·567
Severe gestational hypertension	93/296 (31%)	94/283 (33%)	0·95 (0·75–1·20)	0·644
Early onset pre-eclampsia (<32 weeks' gestation)	37/296 (13%)	38/283 (13%)	0·93 (0·61–1·42)	0·740
Severe pre-eclampsia	52/296 (18%)	60/283 (21%)	0·83 (0·59–1·16)	0·268
Moderately severe thrombocytopenia	13/63 (21%)	12/74 (16%)	1·27 (0·63–2·59)	0·505
Uric acid greater than reference values for gestational age	22/27 (82%)	19/24 (79%)	1·03 (0·78–1·35)	0·835
Renal failure (creatinine >120 mmol/L)	7/58 (12%)	5/68 (7%)	1·64 (0·55–4·90)	0·369
Liver failure	8/53 (15%)	7/63 (11%)	1·36 (0·53–3·50)	0·524
Eclampsia‡	4/296 (1%)	5/283 (2%)	0·76 (0·21–2·82)	0·747
Placental abruption	9/295 (3%)	5/283 (2%)	1·73 (0·59–5·09)	0·420
Pulmonary oedema	0/296 (0%)	1/283 (0%)	0	0·489
Stroke	0/296 (0%)	0/283 (0%)	..	..
ICU admission >24 h	2/296 (1%)	3/283 (1%)	0·64 (0·11–3·79)	0·680
HELLP syndrome	10/69 (15%)	7/81 (9%)	1·68 (0·67–4·17)	0·260
Maternal death*	2/323 (1%)	2/310 (0%)	0·96 (0·14–6·77)	1·00
Participant hospital stay 7 days or more after birth	23/292 (8%)	13/282 (5%)	1·71 (0·88–3·31)	0·107
Caesarean section	176/295 (60%)	152/283 (54%)	1·11 (0·96–1·28)	0·149
Birthweight <2500 g	79/264 (30%)	73/243 (30%)	1·00 (0·76–1·30)	0·977
Preterm birth (<37 weeks' gestation)	112/296 (38%)	119/283 (42%)	0·90 (0·74–1·10)	0·301
Early preterm birth (<32 weeks' gestation)	47/296 (16%)	57/283 (20%)	0·79 (0·56–1·12)	0·182
Apgar score <7 at 5 min	5/255 (2%)	11/239 (5%)	0·43 (0·15–1·21)	0·097
Perinatal death or admission to neonatal ICU for 24 h or more	52/265 (20%)	43/243 (18%)	1·11 (0·77–1·60)	0·578
Stillbirth	27/296 (9%)	33/283 (12%)	0·78 (0·48–1·27)	0·316
Pregnancy loss, stillbirth, or neonatal death before discharge*	65/323 (20%)	76/309 (25%)	0·82 (0·61–1·10)	0·177
Pregnancy loss, stillbirth, or neonatal death before 6 weeks*	74/323 (23%)	80/309 (26%)	0·88 (0·67–1·16)	0·383
Severe pre-eclamptic complications index§	106/296 (36%)	103/283 (36%)	0·98 (0·79–1·22)	0·884
Severe maternal morbidity and mortality index¶	63/296 (21%)	65/283 (23%)	0·93 (0·68–1·26)	0·625

RR=risk ratio. ICU=intensive care unit. HELLP=haemolysis, elevated liver enzymes, and low platelet count. The denominator for all outcomes includes pregnant participants who reached ≥20 weeks' gestation, with the exception of outcomes denoted by * or †.

* The denominator includes participants that became pregnant during the study period, excluding those who requested an abortion (five in each group), withdrew (one in each group), or were lost to follow-up before 20 weeks' gestation (one in calcium group and four in placebo group).

† The denominator includes all randomised participants.

‡ All participants with eclampsia also had pre-eclampsia.

§ Severe pre-eclamptic complications index includes any of the following: severe pre-eclampsia, early-onset pre-eclampsia, eclampsia, placental abruption, HELLP syndrome, or severe gestational hypertension.

¶ Severe maternal morbidity and mortality index includes any of the following: admission to ICU or any special care unit, eclampsia, placental abruption, HELLP syndrome, renal failure, or death.

**Table 5:** Per-protocol analysis of the primary outcome for participants with >80% compliance

	**Calcium (n/N [%])**	**Placebo (n/N [%])**	**Risk ratio**	**95% CI**	**p value**
**Compliance >80% from randomisation up to the last PPV**					
Pre-eclampsia*	20/98 (20%)	33/100 (33%)	0·62 (0·38–1·00)	..	0·045
**Compliance >80% from last PPV to the 20-week visit**					
Pre-eclampsia†	30/144 (21%)	47/149 (32%)	0·66 (0·44–0·98)	..	0·037
**Compliance >80% from randomisation up to the last PPV and compliance >80% from last PPV to the 20-week visit**					
Pre-eclampsia	14/69 (20%)	23/72 (32%)	0·64 (0·36–1·13)	..	0·116

PPV=prepregnancy visit.

* 98 in the calcium group, because one participant was not eligible and one was lost to follow-up.

† 144 in the calcium group because one participant was not eligible.

Serious adverse events were systematically recorded. Most were classified as unrelated to the intervention, none were related or likely related, and in five of 82 women in the calcium group and 12 of 79 in the placebo group, a serious adverse event was classified as unlikely related. 12 participants died during the 6-year trial period (five calcium, seven placebo; appendix). Of these participants, eight did not become pregnant and four died within 6 weeks of childbirth (two calcium, two placebo). One death (in the placebo group) was associated with hypertension. We were informed of one further death outside of the trial period (more than 6 weeks after completion of a pregnancy). No deaths were trial-related.

## Discussion

This trial does not show a significant reduction in recurrent pre-eclampsia with calcium supplementation before and during early pregnancy. This trial is fundamentally different from previous trials of calcium to prevent pre-eclampsia. We sought a specific effect of calcium in early pregnancy (when defective placentation is thought to initiate the pre-eclampsia pathway) on the evolution of pre-eclampsia in late pregnancy despite high-dose calcium supplementation from 20 weeks' gestation in both study groups, as recommended by WHO.^22^ The trial was designed to detect a 40% reduction in pre-eclampsia and the results do not show a large effect size. Possible reasons include the following: there is no real effect; there is a real effect but it is smaller than the 40% for which the trial was powered, because of a type 2 statistical error (of which there was a 20% risk); or because suboptimal compliance diluted the effect. The finding of a greater reduction in pre-eclampsia with calcium supplementation in the per-protocol analysis for those participants with compliance of more than 80% suggests that suboptimal compliance might have played a part.

The composite secondary outcome pre-eclampsia or pregnancy loss, or both, merits discussion because pregnancy loss and pre-eclampsia might have an association. This outcome might overcome confounding of pre-eclampsia findings by an early effect of calcium on pregnancy loss, and might be a better indicator of the overall effect of calcium supplementation. With calcium supplementation, we found an 18% reduction in this outcome (95% CI 0–34), which was of borderline statistical significance (p=0·050).

The numbers of serious outcomes were too small for meaningful statistical analysis. The low prevalence of severe complications is to be expected, as both groups received high-dose calcium from 20 weeks' gestation, which is known to reduce the severe complications of pre-eclampsia. The high quality of care received as a result of participation in the trial might also have contributed to reducing poor outcomes, as illustrated by the fact that, despite there being a high prevalence of previous eclampsia (19%) in the overall sample, only 1·4% of participants in the calcium group and 1·8% in the placebo group developed eclampsia during the trial.

The intervention in our study was purposefully of a low intensity: a low dose of calcium (500 mg daily) was used because a higher dose, even if effective, would not be implementable on a broad scale, such as through food fortification. The randomised intervention stopped at 20 weeks' gestation, thus the effect was limited to prepregnancy and early pregnancy calcium effects, avoiding confounding by the known beneficial effects of calcium later in pregnancy. Our study would thus have underestimated the overall effect of calcium supplementation throughout pregnancy, which could be extrapolated to be the summation of any effects of supplementation before and in early pregnancy plus the effects of supplementation after 20 weeks as measured by previous trials.^17^

To our knowledge, this is the first trial attempting to measure the effects of calcium supplementation before conception and measuring the effect on pregnancy outcomes. Despite the challenges of this type of trial, the research team managed to recruit and retain sufficient participants over a long period of time. The strategies put in place leading to this success have been described elsewhere.^23^ One previous study of calcium supplementation in early pregnancy has been reported.^27^ This small RCT evaluated calcium and antioxidant supplementation commenced at 8–12 weeks' gestation compared with placebo and found reductions in pre-eclampsia (two of 29 in the calcium group *vs* nine of 31 in the placebo group) and miscarriage (zero of 29 *vs* eight of 31) with the intervention; the results are suggestive of a calcium effect because antioxidants have not been found to reduce pre-eclampsia.^28^

During recruitment, the diagnosis of previous pre-eclampsia was in accord with the hospital records in most cases. However, if the record was not available, we relied on the information reported by participants in this respect. Although we are confident that women in these setting are aware of their diagnosis when they have pre-eclampsia or eclampsia, we cannot exclude the possibility of errors. Such errors might have diluted the level of risk in both groups but would not affect the comparability of the groups.

The study groups were comprised of participants from a population with generally low dietary calcium intake and, therefore, the effects of calcium supplementation might not be generalisable to women with adequate dietary intake. In addition, the study participants were at high risk of pre-eclampsia on the basis of their having a previous diagnosis of pre-eclampsia. A general obstetric population would include women at varying and often unpredictable risk of pre-eclampsia.

Calcium supplementation commenced before pregnancy until 20 weeks' gestation, compared with placebo, did not show a significant reduction in recurrent pre-eclampsia. As the trial was powered to detect a large effect size, we cannot rule out a small to moderate effect of this intervention.

Calcium supplementation is recommended by WHO from 20 weeks of pregnancy at doses of 1·5–2·0 g per day, particularly in a population with low calcium intake.^22^ Dietary calcium intake in low-income settings is typically exceptionally poor.^16^ The cost of one tablet daily (600 mg) is estimated to cost under US$8 per person per year.^29^ Findings from our study, with appropriate recognition of the absence of significance, should be considered together with all other available evidence on the benefits of adequate calcium intake for pregnant women and the general population, in determining the cost and benefits of strategies to improve calcium intake at a population level.

The findings of this complex and lengthy trial suggest that a larger trial would be required to adequately test a small to moderate effect of calcium on pre-eclampsia. Researchers and policymakers will need to evaluate whether further research, possibly using a population-based approach, is justified.

## Data sharing

The authors support data sharing and queries in this regard can be addressed to the contact author.
