# Supplementary material for: Prepregnancy and early pregnancy calcium supplementation among women at high risk of pre-eclampsia: a multicentre, double-blind, randomised, placebo-controlled trial
Source: Lancet. 2019 Jan 26;393(10169):330–9. doi: 10.1016/S0140-6736(18)31818-X (PMC6346082; doi:10.1016/S0140-6736(18)31818-X)
Supplement: Supplementary appendix [file mmc1.pdf]

THE LANCET

Supplementary appendix

This appendix formed part of the original submission and has been peer reviewed. We post it as supplied by the authors.

Supplement to: Hofmeyr GJ, Betrán AP, Singata-Madliki M, et al. Prepregnancy and early pregnancy calcium supplementation among women at high risk of pre-eclampsia: a multicentre, double-blind, randomised, placebo-controlled trial. *Lancet* 2019; **393:** 330–39.

Supplementary Appendix

Pre-pregnancy and early pregnancy calcium supplementation among women at high risk of pre-eclampsia: a multicentre, double-blind randomised, placebo-controlled trial

1. Description of the composition of the participants by country

Table 1a. All participants at trial entry by country (n=1355)

			Treatment Group		Total
			Placebo	Calcium	
Country	Argentina	n	57	60	117
		%	8·4%	8·8%	8·6%
	Zimbabwe	n	141	141	282
		%	20·8%	20·8%	20·8%
	South Africa	n	479	477	956
		%	70.8%	70.4%	70·6%
Total			677	678	1355

Table 1b. Pregnant vs not pregnant by country (n=1355)

			Country			Total
			Argentina	Zimbabwe	South Africa	
Pregnant	No	n	84	104	516	704
		%	71·8%	36·9%	54·0%	52·0%
	Yes	n	33	178	440	651
		%	28·2%	63·1%	46·0%	48·0%
Total			117	282	956	1355

Table 1c. Participants with pregnancy beyond 20 weeks' gestation by country (n=581)

			Treatment Group		Total
			Placebo	Calcium	
Country	Argentina	n	12	14	26
		%	4·2%	4·7%	4·5%
	Zimbabwe	n	77	83	160
		%	27·2%	27·9%	27·5%
	South Africa	n	194	201	395
		%	68·6%	67·4%	68·0%
Total			283	298	581

Table 1d. Participants with pre-eclampsia according to treatment group - Argentina

Argentina					
			Treatment Group		Total
			Placebo	Calcium	
Pre-eclampsia	No	n	10	12	22
		%	83·3%	85·7%	84·6%
	Yes	n	2	2	4
		%	16·7%	14·3%	15·4%
Total		n	12	14	26
		%	100·0%	100·0%	100·0%

Table 1e. Participants with pre-eclampsia according to treatment group - Zimbabwe

Zimbabwe					
			Treatment Group		Total
			Placebo	Calcium	
Pre-eclampsia	No	n	71·4%	75·6%	73·6%
		%	22	20	42
	Yes	n	28·6%	24·4%	26·4%
		%	77	82	159
Total		n	12	100·0%	100·0%
		%	100·0%	71·4%	75·6%

Table 1f. Participants with pre-eclampsia according to treatment group - South Africa

South Africa					
			Treatment Group		Total
			Placebo	Calcium	
Pre-eclampsia	No	n	136	153	289
		%	70·1%	76·5%	73·4%
	Yes	n	58	47	105
		%	29·9%	23·5%	26·6%
Total		n	194	200	394
		%	100·0%	100·0%	100·0%

2. Follow-up time of participants in the trial

Table 2. Duration of follow up of participants randomised

Treatment Group			Statistic	Standard Error
Placebo (n=677)	Mean (months)		29·1	0·791
	95% CI	Inferior limit	27·6	
		Superior limit	30·7	
	Median		27·0	
	Variance		223·7	
	Standard deviation		15·0	
	Minimum		2·0	
	Maximum		68·0	
	Range		66·0	
	Interquartile range		21·0	
	Asymmetry		0·493	0·129
	Kurtosis		-0·479	0·257
Calcium (n=678)	Mean (months)		28·3	0·787
	95% CCI	Inferior limit	26·8	
		Superior limit	29·9	
	Median		27·0	
	Variance		215·0	
	Standard deviation		14·7	
	Minimum		2·0	
	Maximum		67·0	
	Range		65·0	
	Interquartile range		20·0	
	Asymmetry		0·444	0·131
	Kurtosis		-0·399	0·261
